# Association and functional analysis of angiotensin-converting enzyme 2 genetic variants with the pathogenesis of pre-eclampsia

**DOI:** 10.3389/fendo.2022.926512

**Published:** 2022-11-07

**Authors:** Gongchen Huang, Yukun Wang, Linyuan Qin, Bo Huang, Xiangyuan Yu

**Affiliations:** ^1^ Guangxi Key Laboratory of Environmental Exposomics and Entire Lifecycle Heath, Guangxi Health Commission Key Laboratory of Entire Lifecycle Health and Care, School of Public Health, Guilin Medical University, Guilin, China; ^2^ Scientific Experiment Center, Guilin Medical University, Guilin, China; ^3^ Institute of Preventive Medicine, School of Public Health, Guilin Medical University, Guilin, China

**Keywords:** angiotensin-converting enzyme 2, variation, pre-eclampsia (PE), risk, functional analysis

## Abstract

**Objective:**

The aim of this study was to investigate the relationship between potential functional single-nucleotide polymorphisms (SNPs) of the angiotensin-converting enzyme 2 (*ACE2*) gene and the pathogenesis of pre-eclampsia (PE) in Guangxi, China.

**Materials and methods:**

A case–control study was conducted involving 327 PE cases and 591 age-matched, normal, singleton pregnant women. Potential functional *ACE2* gene variants (rs2106809 A>G, rs6632677 G>C, and rs2074192 C>T) were selected and genotyped using kompetitive allele-specific PCR. The strength of the associations between the studied genetic variants and the risk of PE were evaluated using odds ratios (ORs) and corresponding 95% confidence intervals (CIs).

**Result:**

After adjusting for age and body mass index (BMI), unconditional logistic regression analysis showed that rs2106809 A>G was significantly associated with PE risk (AG *vs*. AA, OR = 1.43, 95% CI = 1.03–1.99, *p* = 0.034; AG/GG *vs*. AA, OR = 1.45, 95% CI = 1.06–1.99, *p* = 0.019), especially with severe PE (AG *vs*. AA, adjusted OR = 1.70, 95% CI = 1.10–2.61; AG/GG *vs*. AA, adjusted OR = 1.71, 95% CI = 1.14–2.57). Further stratified analysis showed that rs2106809 was even more pronounced in subjects in the pre-pregnancy BMI (pre-BMI) >23 kg/m^2^ (adjusted OR = 2.14, 95% CI = 1.32–3.45) and triglyceride (TG) >2.84 mmol/L subgroups (adjusted OR = 1.81, 95% CI = 1.09–3.01) under the dominant genetic model. We also found that rs2106809 interacted with pre-BMI (*p*_interaction_ = 0.040), thereby affecting an individual’s genetic susceptibility to PE. Multiple dimension reduction analysis demonstrated that rs2106809 made the best one-locus model, and the three-locus model was the best interaction model for predicting PE risk. Functional analysis suggested that rs2106809 A>G causes a change in the reliability of classifications of two putative splice sites in the *ACE2* gene, potentially regulating the expression of functional genes (*PIR*, *ACE2*, and *CLTRN*) in multiple tissues and cell lines (*p<* 0.05).

**Conclusion:**

The *ACE2* gene rs2106809 A>G variant is significantly associated with the risk of PE *via* individual locus effects and/or complex gene–gene and gene–environment interactions. Regulating the expression of functional genes such as *PIR*, *ACE2*, and *CLTRN* may be the molecular mechanism by which rs2106809 increases an individual’s susceptibility to PE.

## Introduction

Pre-eclampsia (PE), a common disease that occurs during pregnancy, has a global incidence of 3%–5% and approximately 2.9% in China (1). PE is characterized by hypertension (140/90 mmHg) and albuminuria (0.3 g/day) after 20 weeks of pregnancy. In recent years, the incidence of PE has increased, especially in developing countries (2). Clinical studies have shown that PE can cause systemic small vessel spasms, endothelial injury, and a decrease in blood perfusion of various systems and organs caused by local ischemia, which can injure the mother and fetus. As the disease progresses, it may even lead to terminal organ dysfunction and multiple severe adverse pregnancy outcomes, such as maternal eclampsia, oligohydramnios, placental abruption, hemolysis, elevated liver enzymes, and low platelet count (HELLP) syndrome, and cardiovascular disease or fetal intrauterine growth restriction, premature birth, and fetal perinatal death (3, 4). PE seriously endangers both the short-term and long-term health of mothers and infants. However, details regarding the etiological mechanism of PE remain unclear.

PE may be affected by conditions such as advanced pregnancy, chronic hypertension, inflammatory immune overactivation, vascular endothelial cell damage, nutritional deficiency or obesity, and insulin resistance (5). In addition, previous studies have found that those with an individual or family history of PE are significantly more likely to develop PE during pregnancy (6, 7). Thus, in addition to various environmental factors, the risk of PE is also regulated by genetic factors. Single-nucleotide polymorphisms (SNPs) as DNA sequence variations caused by the conversion or transversion of a single nucleotide are the most common genetic variations in humans (8). They play important roles in the genetic anatomy of complex traits and diseases, and are useful for identifying population-based susceptibility genes (9). Genome-wide association and case–control studies have identified a series of susceptibility genes and susceptibility loci related to the risk of PE, including the zinc finger protein 831(ZNF831) gene polymorphism rs259983, FTO alpha-ketoglutarate dependent dioxygenase (FTO) rs1421085, MDS1 and EVI1 complex locus (MECOM) rs1918975, angiotensinogen (AGT) M235T, storkhead box 1 (STOX1) Y153H, and interleukin 6 (IL-6) 176 G>C (10–13).

Studies have confirmed that the renin–angiotensin system (RAS) is involved in vascular remodeling and blood pressure regulation during pregnancy (14–17). The *ACE2* gene plays a key role in the RAS system. ACE2 can induce angiotensin II (Ang II) to produce angiotensin 1–7 (Ang1–7), which dilate blood vessels and thereby lower body blood pressure (18). Tamanna et al. found that the regulation of *ACE2* plays an important role in PE and fetal growth restriction, suggesting that *ACE2* could be a new target for the treatment and/or prevention of pregnancy complications (19). Several *ACE2* gene variants, such as rs2074192, rs2106809, rs2048683, and rs4240157, are reportedly significantly associated with the risk of hypertension (20, 21). Based on current research evidence, we hypothesized that the *ACE2* gene and its variants play a key role in regulating the incidence and outcome of various pregnancy complications, including PE.

To date, no studies have been published detailing the relationship between *ACE2* gene variants and susceptibility to PE. We therefore conducted a case–control study involving 327 PE patients and 591 healthy pregnant women to explore the associations between three candidate variants in the *ACE2* gene (rs2106809 A>G, rs6632677 G>C, and rs2074192 C>T) and the pathogenesis of PE.

## Materials and methods

### Study population

A total of 918 subjects with 327 cases with PE (118 women with mild PE and 61 women with severe PE) and 591 controls were recruited from the First Affiliated Hospital of Guilin University of Medical Sciences between September 2014 and April 2016. Participants who met the diagnostic criteria for PE (first presentation after 20 weeks of gestation with systolic blood pressure ≥140 mmHg or diastolic blood pressure ≥90 mmHg and urine protein ≥0.3 g/24 h) were included as PE cases. Age-matched (± 3 years) healthy pregnant women were recruited during the same period. All subjects were singleton pregnancies and unrelated. Pregnant women with diabetes, hypertension, congenital genetic diseases, cardiovascular diseases, or other pregnancy-related diseases were excluded. Study participants signed an informed consent form before the study, and the Ethics Committee Review Board of Guilin Medical University and the Affiliated Hospital of Guilin Medical University approved the study protocol.

### Clinical and biochemical data

Clinical and biological characteristics were obtained from unified questionnaires and patient medical records, including weight, height, pre-pregnancy BMI (pre-BMI), diastolic blood pressure (DBP), systolic blood pressure (SBP), fasting plasma glucose (FPG), triglyceride (TG), total cholesterol (TC), and high-density lipoprotein cholesterol (HDL-c).

### Genomic DNA extraction

The genomic DNA was extracted from EDTA-treated whole blood using a DNA extraction kit (Aidlab Biotechnologies Co., Ltd., China) and stored in a refrigerator at −20°C prior to PCR.

### SNP selection

Potential functional SNPs of the *ACE2* gene were screened using the NCBI dbSNP database (http://www.ncbi.nlm.nih.gov/projects/SNP) and SNPinfo Web Server (http://snpinfo.niehs.nih.gov/). The minor allele frequency (MAF) of candidate SNPs reported in HapMap-based Han Chinese in Beijing (HCB) was >0.05, and the linkage disequilibrium (LD) coefficient between SNPs was<0.8 (*r* ^2^< 0.8). Finally, three genetic variants (rs2106809 A>G, rs6632677 G>C, and rs2074192 C>T) located in the *ACE2* gene were selected for further study.

### SNP genotyping using the kompetitive allele-specific PCR method

**Figure 1:**
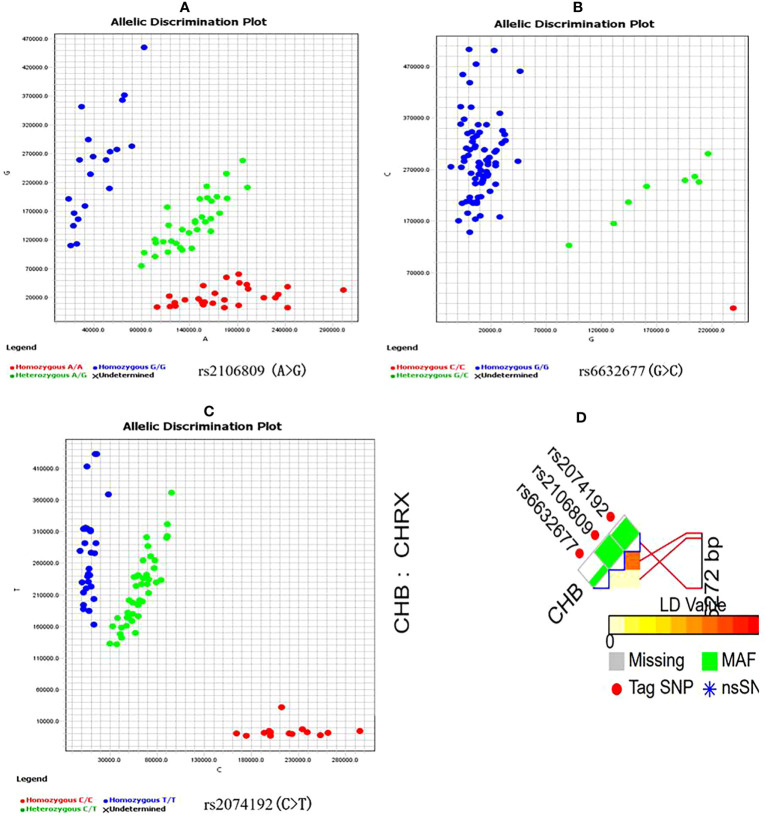
ACE2 gene candidate genetic variations selection and genotyping **(A–C)** are genotyping scatter plot of rs2106809 A>G, rs6632677G>C and rs2074192C>T respectively; **(D)** is ACE2 fiunctional SNPs selection by SNPinfo Web Server online tool, LD: r2<0.8.

Candidate *ACE2* gene variants were genotyped using a kompetitive allele-specific PCR (KASP) method, and the corresponding specific PCR primers were designed and synthesized by Sangon Biotech Co., Ltd. (Shanghai, China) (**Table 1**). Reactions (10 µl each) were carried out in a 96-well plate and included 5 µl of FLu-Arms 2× PCR mix, 0.5 µl of 10 μM each of three specific primers (F1: 0.1 µl, F2: 0.1 µl, and R: 0.3 µl), 0.5 µl (25–150 ng) of DNA, and 4 µl of ddH_2_O. Two allele-specific forward primers were labeled with the respective fluorochromes FAM and HEX. Reactions were performed according to the following standard KASP-PCR program: pre-denaturation at 95°C for 3 min, followed by 10 touchdown cycles of 95°C for 15 s (denaturation), 61–55°C for 60 s (annealing and elongation), and 30 cycles of 95°C for 15 s and 55°C for 60 s, with a final incubation at 30°C for 30 s. **Figure 1** shows a genotyping scatter plot of the candidate loci.

**Table 1:** The information of primer sequences for selected SNPs.

Primer name	Primer sequence
rs2106809-F1	5' -GGAGGTGACCAAGTTCATGCTTTTCCATATCTCTATCTGATGGA- 3'
rs2106809-F2	5' - GAAGGTCGGAGTCAACGGATTTTCCATATCTCTATCTGATGGG- 3'
rs2106809-R	5' -TAAAGCTGCTGATGTAGAGATGTGGAG - 3'
rs6632677-F1	5' -GAAGGTGACCAAGTTCATGCTAGAGACCATAGCTCTAGCCAG- 3'
rs6632677-F2	5' -GAAGGTCGGAGTCAACGGATTAGAGACCATAGCTCTAGCCAC- 3'
rs6632677-R	5' -CTTCTAAGTCTCATATGGTTGCATTC- 3'
rs2074192-F1	5' -GAAGGTGACCAAGTTCATGCTGGAAATGTATAAATGGTTGGC- 3'
rs2074192-F2	5' -GAAGGTCGGAGTCAACGGATTTGGAAATGTATAAATGGTTGGT- 3'
rs2074192-R	5' -CTTCTAAGTCTCATATGGTTGCATTC-3'

F, the forward primer; R, the reverse primer.

### Statistical analysis

Statistical analysis was performed with IBM SPSS Statistics 28 for Windows (IBM Corp., Armonk, NY, USA). Clinical and biochemical features were compared by calculating the mean ± standard deviation ( $\bar{\text{x}}$ ± sd). Chi-square (*χ* ^2^) test was used to assess the differences of genotypes between cases and controls. A *χ* ^2^ goodness-of-fit test was performed to determine whether the distribution about genotypes of SNPs in controls conformed to Hardy–Weinberg equilibrium (HWE). Logistic regression model was used to evaluate the associations between genotypes of each variant with PE by computing the odds ratios (ORs) and their 95% confidence intervals (CIs). A *p*-value< 0.05 was considered as statistically significant and all statistical tests were two-tailed.

In addition, the false-positive reporting probability (FPRP) was estimated using the method described by Wacholder et al. (22) to assess the robustness of the statistically significant associations found. The threshold was set to 0.2 and the prior probability was set to 0.1 to detect the noteworthy with an OR value of 1.5 or 0.67, and the *α* level was equal to the observed *p*-value.

The multiple dimension reduction (MDR) software (version 3.0.2) was used to investigate the joint effect of SNPs. A 100-fold cross-validation and 1,000-fold permutation testing were adopted under the null hypothesis of no association. The cross-validation consistency (CVC) and the testing balanced accuracy helped to identify the best model among all possibilities considered (23).

If a significant association between genetic variation and PE risk is detected, the potential function of the variation regulating the putative splice sites will be analyzed by Alternative Splice Site Predictor (ASSP) tool (24). In the ASSP analysis, codon usage and stop codons for all three possible reading frames (F1—frame 1, F2—frame 2, and F3—frame 3) and scores of the preprocessing models reflecting splice site strength are calculated, and the types of putative splice sites are classified by the corresponding backpropagation network with the output activations and the confidence by ASSP analysis. In order to explore the function of significantly associated SNP and possible regulation of related gene expression, we further applied GWAS4D online analysis (http://www.mulinlab.org/gwas4d/) (25) based on GTEx database to analyze expression quantitative trait locus (eQTL) between candidate variants and gene transcription level and clarify the potential molecular regulation mechanism of variant.

## Results

### Characteristics of the study population

A total of 918 participants were selected, including 327 women diagnosed with PE and 591 healthy controls. Analysis of the general population data revealed that the indexes of pre-BMI, SBP, DBP, FPG, TG, and HDL-C were higher among PE cases than controls (*p<* 0.001), as shown in **Table 2**.

**Table 2:** Demographic and clinical characteristics in PE cases and controls ( $\bar{\text{x}}$ ± s).

	Case (n=327)	Controls (n=591)	t	*P*
Age (years)	28.39 ± 4.76	29.20 ± 4.26	-2.55	0.01
Pre-pregnancy BMI (kg/m^2^)	25.39 ± 4.88	22.33 ± 2.72	10.45	<0.001
SBP (mmHg)	145.90 ± 17.37	109.67 ± 9.60	36.226	<0.001
DBP (mmHg)	95.11 ± 13.10	68.7 ± 8.25	32.56	<0.001
Mild pre-eclampsia	187 (0.57)	–	/	/
Severe pre-eclampsia	140 (0.43)	–	/	/
Proteinuria (g/24 h)	4.43 ± 1.93	–	/	/
FPG (mmol/L)	5.41 ± 1.60	4.43 ± 0.37	10.87	<0.001
TG (mmol/L)	3.64 ± 1.74	2.41 ± 1.05	11.64	<0.001
TC (mmol/L)	6.57 ± 1.71	5.23 ± 1.05	12.77	<0.001
HDL-c (mmol/L)	1.89 ± 0.45	1.64 ± 0.41	8.40	<0.001

SBP, systolic blood pressure; DBP, diastolic blood pressure; FPG, fasting plasma glucose.

TG, Triglyceride; TC, Total cholesterol; HDL-c, high density lipoprotein cholesterol.

### Association between studied variants and PE risk

The candidate SNPs were genotyped using the KASP method. The D and *r* ^2^ values were calculated to evaluate the LD of studied SNPs from the genotyping data using the SHEsis online tool, which revealed no significant degree of LD among the three SNPs (data not shown). The genotype frequencies of rs2106809, rs6632677, and rs2074192 among controls obeyed HWE (*χ* ^2^ = 1.08, *p* = 0.299; *χ* ^2^ = 0.69, *p* = 0.405 and *χ* ^2^ = 4.55, *p<* 0.05, respectively). After adjustment for age and pre-BMI, logistic regression analysis showed that the associations remained significant in the heterozygote comparison (AG *vs*. AA, adjusted OR = 1.43, 95% CI = 1.03–1.99, *p* = 0.034) and in the dominant model (AG/GG *vs*. AA, adjusted OR = 1.45, 95% CI = 1.06–1.99, *p* = 0.019). However, compared with AA genotype carriers, the associations in GG genotype carriers exhibited marginal statistical significance (GG *vs*. AA, adjusted OR = 1.50, 95% CI = 0.98–2.32, *p* = 0.063). We failed to find significant associations between *ACE2* rs6632677 C>G and rs2074192 C>T and susceptibility to PE in the present study (*p* > 0.05), as shown in **Table 3**.

**Table 3:** Association between genotypes of selected SNPs and PE risk.

Genotype	Case	Control	*P* ^a^	Crude OR (95%CI)	*P* ^b^	Adjusted OR (95%CI)	*P* ^c^
rs2106809							
AA	121	167	**0.020**	1		1	
AG	153	306		1.45 (1.07~1.96)	**0.017**	1.43 (1.03~1.99)	**0.034**
GG	53	118		1.61 (1.08~2.41)	**0.019**	1.50 (0.98~2.32)	0.063
AG/GG	206	424		1.49 (1.12~1.99)	**0.006**	1.45 (1.06~1.99)	**0.019**
AA/AG	270	473	0.161	1		1	
GG	53	118		1.29 (0.90~1.84)	0.162	1.21 (0.83~1.78)	0.328
rs6632677							
GG	282	515	0.708	1		1	
GC	39	62		0.87 (0.57~1.33)	0.523	0.84 (0.53~1.34)	0.469
CC	6	14		1.28 (0.49~3.36)	0.620	1.28 (0.46~3.56)	0.634
GC/CC	45	76		0.93 (0.62~1.37)	0.699	0.90 (0.59~1.39)	0.903
GG/GC	321	577	0.596	1		1	
CC	6	14		1.30 (0.49~3.41)	0.597	1.31 (0.47~3.62)	0.607
rs2074192							
CC	65	121	0.892	1		1	
CT	150	264		0.95 (0.66~1.36)	0.761	1.01 (0.68~1.49)	0.975
TT	109	209		1.02 (0.69~1.49)	0.938	1.04 (0.69~1.57)	0.847
CT/TT	259	470		0.98 (0.70~1.37)	0.882	1.02 (0.71~1.47)	0.910
CC/CT	215	385	0.712	1		1	
TT	109	206		1.06 (0.79~1.41)	0.712	1.04 (0.76~1.41)	0.820

a Two-sided χ^2^ test for genotypes distributions between cases and controls.

b Unconditional logistic regression analysis.

c Adjusted for age, pre-BMI in logistic regression models. CI, confidence interval. OR, odds ratio. Pa for allele frequencies in cases and controls using 2-sided χ2 test. Pb and Crude OR (95%CI) obtained using Unconditional logistic regression analyses. Pc and adjusted OR (95%CI) adjusted by age and pre-BMI using logistic regression. Bold indicates statistical significance (P < 0.05).

We also estimated the effect of rs2106809 A>G on PE risk using a stratified analysis under the dominant genetic model. After adjusting for age and pre-BMI, this association remained in the pre-BMI >23 kg/m^2^ subgroup (adjusted OR = 2.14, 95% CI = 1.32–3.45) and the TG >2.84 mmol/L subgroup (adjusted OR = 1.81, 95% CI = 1.09–3.01). However, using the same method, a stratified analysis shows that compared with the AA genotype, the AG/GG genotype exhibited a marginal statistical association with PE among subjects ≤29 years of age (adjusted OR = 1.46, 95% CI = 0.97–2.19, *p* = 0.067) and those with TC >5.71 mmol/L (adjusted OR = 1.53, 95% CI = 0.96–2.44, *p* = 0.074). In the dominant genetic model, we also found that rs2106809 interacted with pre-BMI (*p*_interaction_ = 0.040) to affect susceptibility to PE, as shown in **Table 4**.

**Table 4:** Stratification analysis for associations between ACE2 rs2106809 A>G and PE risk.

Variables	AA (Case/Control)	AG/GG (Case/Control)	Crude OR (95%CI)	*P* ^a^	Adjusted OR (95%CI)	*P* ^b^	*P* ^c^
Age (years)							0.195
≤ 29	81/106	125/236	**1.44 (1.01-2.07)**	**0.047**	1.46 (0.97-2.19)	0.067	
> 29	40/61	80/188	1.54 (0.96-2.48)	0.076	1.43 (0.86-2.38)	0.173	
Pre-BMI (kg/m^2^)							**0.040**
≤ 23	35/119	83/289	1.02 (0.65-1.61)	0.917	0.95 (0.60-1.51)	0.833	
> 23	86/48	123/135	**1.97 (1.28-3.02)**	**0.002**	**2.14 (1.32-3.45)**	**0.002**	
FPG (mmol/L)							0.865
≤ 4.78	51/145	89/367	1.45 (0.98-2.15)	0.065	1.47 (0.95-2.23)	0.086	
> 4.78	69/22	116/57	1.54 (0.87-2.74)	0.14	1.47 (0.81-2.67)	0.201	
TG (mmol/L)							0.332
≤ 2.84	46/128	79/300	1.37 (0.90-2.07)	0.145	1.34 (0.86-2.09)	0.198	
> 2.84	73/39	125/124	**1.86 (1.17-2.95)**	**0.009**	**1.81 (1.09-3.01)**	**0.022**	
TC (mmol/L)							0.978
≤ 5.71	39/118	63/294	1.54 (0.98-2.43)	0.061	1.52 (0.93-2.49)	0.098	
> 5.71	81/48	141/130	**1.56 (1.01-2.39)**	**0.044**	1.53 (0.96-2.44)	0.074	
HDL-c (mmol/L)							0.704
≤ 1.72	42/98	76/269	1.52 (0.98-2.36)	0.065	1.25 (0.79-2.04)	0.372	
> 1.72	77/69	128/155	1.35 (0.91-2.02)	1.141	1.51 (0.98-2.33)	0.061	

a Unconditional logistic regression analysis.

b Adjusted for age, pre-BMI in logistic regression models.

c Test for multiplicative interaction obtained from logistic regression models. CI, confidence interval. OR, odds ratio. Pa and Crude OR (95%CI) obtained using unconditional logistic regression analyses. Pb and adjusted OR (95%CI) adjusted by age and pre-BMI using logistic regression. Pc for multiplicative interaction tests obtained from logistic regression models. Bold indicates statistical significance (P < 0.05).

### Association between genotypes of selected SNPs and PE severity

There were significant associations observed between *ACE2* rs2106809 and severe PE under the heterozygote comparison (AG *vs*. AA, adjusted OR = 1.70, 95% CI = 1.10–2.61, *p* = 0.017) and the dominant model (AG/GG *vs*. AA, adjusted OR = 1.71, 95% CI = 1.14–2.57, *p* = 0.009). Furthermore, the rs2106809 GG genotype had no association with severe PE compared with the AA genotype but with marginal *p*-value (*p* = 0.052). However, no significant association was found between this variant and the onset of mild PE, *p* > 0.05. We also did not detect a significant association between variants of *ACE2* rs6632677 and rs2074192 with PE risk of different severity (**Table 5**).

**Table 5:** Association between genotypes of selected SNPs and PE risk of different severity.

Genotype	Control (n=591)	Mild PE (n=187)	Severe PE (n=140)	Mild PE vs. control		Severe PE vs. control		Severe PE vs. mild PE	
				ad OR (95% CI)	*P* ^c^ value	ad OR (95% CI)	*P* ^c^ value	ad OR (95% CI)	*P* ^c^ value
rs2106809									
AA	167	64	57	1		1		1	
AG	306	91	62	1.30 (0.86-1.95)	0.216	1.70 (1.10-2.61)	0.017	1.31 (0.81-2.12)	0.272
GG	118	32	21	1.35 (0.80-2.29)	0.264	1.78 (0.99-3.19)	0.052	1.38 (0.71-2.67)	0.338
AG/GG	424	123	83	1.31 (0.89-1.93)	0.17	1.71 (1.14-2.57)	0.009	1.33 (0.84-2.09)	0.221
AA/AG	473	155	119	1		1		1	
GG	118	32	21	1.15 (0.72-1.84)	0.594	1.31 (0.77-2.22)	0.322	1.19 (0.65-2.17)	0.575
rs6632677									
GG	515	162	120	1		1		1	
GC	62	23	16	0.77 (0.44-1.36)	0.373	0.85 (0.46-1.56)	0.593	1.07 (0.54-2.11)	0.857
CC	14	2	4	1.83 (0.39-8.51)	0.44	0.82 (0.25-2.67)	0.745	0.37 (0.07-2.03)	0.249
GC/CC	76	25	20	0.87 (0.51-1.48)	0.606	0.84 (0.48-1.47)	0.543	0.92 (0.49-1.74)	0.807
GG/GC	577	185	136	1		1		1	
CC	14	2	4	1.88 (0.41-8.75)	0.418	0.84 (0.26-2.71)	0.768	0.36 (0.07-2.01)	0.245
rs2074192									
CC	121	37	28	1		1		1	
CT	264	90	60	0.93 (0.57-1.50)	0.762	1.06 (0.66-1.87)	0.706	1.11 (0.61-2.01)	0.732
TT	206	59	50	1.09 (0.65-1.81)	0.755	1.00 (0.58-1.71)	0.996	0.89 (0.48-1.66)	0.715
CT/TT	470	149	110	0.99 (0.63-1.55)	0.97	1.03 (0.64-1.68)	0.896	1.01 (0.58-1.76)	0.969
CC/CT	385	127	88	1		1		1	
TT	123	59	50	1.14 (0.78-1.68)	0.502	0.93 (0.62-1.40)	0.735	0.83 (0.52-1.32)	0.43

c Adjusted for age and pre-BMI in logistic regression models.

### False-positive reporting probability analysis

The FPRP test was adopted to assess the noteworthiness of the observed significant associations between the studied rs2106809 A>G variant and PE risk. A prior probability value of 0.1 and a relatively stringent FPRP cutoff value of 0.2 were set. The FPRP value was 0.19 for the association between rs2106809 and PE risk in the pre-BMI >23 kg/m^2^ subgroup, suggesting that the above associations identified in the present study are noteworthy, as shown in **Table 6**.

**Table 6:** FPRP analysis for the significant associations of the rs2106809 A>G and PE risk.

Comparisons	Adjusted OR (95%CI)	Prior probability					
		0.25	0.1	0.01	0.001	0.0001	0.00001
AG vs. AA	1.43 (1.03-1.99)	0.143	0.333	0.846	0.982	0.998	1.000
AG/GG vs. AA	1.45 (1.06-1.99)	0.092	0.232	0.769	0.971	0.997	1.000
Subgroup							
Pre-BMI>23kg/m^2^	2.14 (1.32-3.45)	0.072	**0.19**	0.72	0.963	0.996	1.000
TG>2.84 mmol/L	1.81 (1.09-3.01)	0.22	0.459	0.903	0.989	0.999	1.000

Bold values indicate that the difference is statistically significant at the test level of α=0.2.

### High-order interaction with PE risk by MDR analysis

The MDR results list the three best possible interaction models. For the one-locus model, it had a training sample accuracy of 0.5437, a testing balanced accuracy of 0.5437, a maximum CVC of 100/100, and a *p*-value of 0.0062. For the three-locus model, which included rs2106809, rs6632677, and rs2074192, it had a training sample accuracy of 0.5785, a testing balanced accuracy of 0.5641, a maximum CVC of 100/100, and a *p*-value<0.001, and suggests that there are interactions among the three loci of the selected ACE2 gene that may synergistically enhance the susceptibility to PE in Chinese Han women, thereby increasing the risk of PE (**Table 7**).

**Table 7:** MDR analysis for the PE risk prediction.

Best model	Training balanced accuracy	Testing balanced accuracy	CVC	χ^2^	*P*
1	0.5437	**0.5437**	100/100	7.48	**0.0062**
1, 2	0.5677	0.5387	100/100	15.55	**< 0.0001**
1, 2, 3	0.5785	**0.5641**	100/100	20.81	**< 0.0001**

Labels: 1. rs2106809; 2. rs6632677; 3. rs2074192.

P-value for 1000-fold permutation test.

The best model was selected as the one in boldface with the maximum prediction precision and the cross-validation consistency (CVC).

### Analysis of the potential regulatory function of rs2106809 A>G

**Figure 2:**
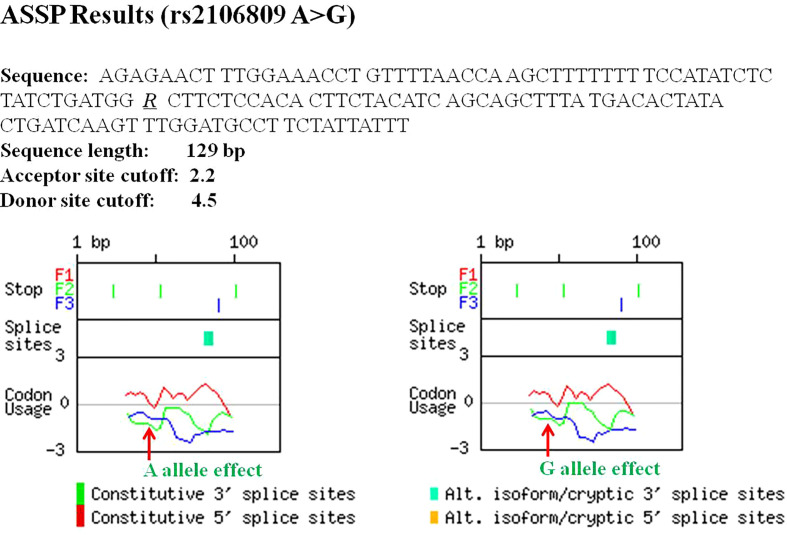
The effect of rs2106809 on alternative splicing sites of ACE2 gene evaluated by the Alternative Splice Site Predictor (ASSP) tool.

ASSP online analysis suggested that the rs2106809 A>G variant causes a change in the reliability of classifications of two putative splice sites (positions 82 bp and 85 bp), with alt. isoform/cryptic activations of 0.811 and 0.818 and constitutive values of 0.183 and 0.174 for the rs2106809 A allele and alt. isoform/cryptic activations of 0.806 and 0.813 and constitutive values of 0.188 and 0.180 for the rs2106809 G allele, respectively. Thus, the regulation of putative splice sites may be changed, as shown in **Table 8** and **Figure 2**.

**Table 8:** The effect of rs2106809 A>G on alternative splicing sites of ACE2 gene evaluated by ASSP tool.

Mutation type	Position (bp)	Putative splice site	Sequence	Score*	Activations**		Constitutive	Confidence**
					Intron GC*	Alt. isoform/Cryptic		
rs2106809-A	82	Alt. isoform/cryptic acceptor	tctacatcagCAGCTTTATG	3.321	0.371	0.811	0.183	0.775
	85	Alt. isoform/cryptic acceptor	acatcagcagCTTTATGACA	5.417	0.386	0.818	0.174	0.788
rs2106809-G	82	Alt. isoform/cryptic acceptor	tctacatcagCAGCTTTATG	3.321	0.386	0.806	0.188	0.767
	85	Alt. isoform/cryptic acceptor	acatcagcagCTTTATGACA	5.417	0.4	0.813	0.180	0.779

* Scores of the preprocessing models reflecting splice site strength; Intron GC values correspond to 70 nt of the neighboring intron; ** Activations are output values of the backpropagation networks used for classification, and high values for one class with low values of the other class imply a good classification; Confidence is a simple measure expressing the differences between output activations.

Furthermore, using the GTEx database, which incorporates 127 tissue/cell type–specific epigenomes datasets, the GWAS4D online tool was used to analyze eQTL between rs2106809 and the regulation of gene transcription. This analysis indicated that rs2106809 could potentially regulate the expression of functional genes associated with PE risk in a variety of specific tissues and cells, including *PIR* (total of 48 studies involved), *ACE2* (44 studies involved), and *CLTRN* (collectrin) (46 studies involved), which were identified as eQTL, as shown in **Table 9**.

**Table 9:** The eQTL analysis on rs2106809 T>C and functional gene transcription levels by GWAS4D online tool.

rs^#^ Position	Regulated Gene symbol	No. of experimental studies (*P*-value< 0.05 / total)	Validated tissues and cell lines (*P*-value< 0.05)
chr X: 15618061	*PIR*	32 / 48	Adipose subcutaneous, Adipose visceral omentum, Adrenal gland, Artery aorta, Artery coronary, Artery tibial, Brain amygdala, Brain anterior cingulate cortex BA24, Brain cerebellar hemisphere, Cerebellum, Brain cortex, Brain frontal cortex BA9, Brain spinal cord cervical c-1, Brain substantia nigra, Mammary tissue, EBV transformed lymphocytes, Transformed fibroblasts, Colon transverse, Esophagus- gastroesophageal junction, Esophagus mucosa, Esophagus muscularis, Heart atrial appendage, Lung, Muscle skeletal, Ovary, Sun exposed lower leg, Small intestine terminal ileum, Testis, Thyroid, Uterus, Vagina, Whole blood
chr X: 15618061	*ACE2*	18 / 44	Artery tibial, Brain amygdala, Brain Anterior cingulate cortex BA24, Brain caudate basal ganglia, Brain cerebellar hemisphere, Cerebellum, Brain cortex, Brain frontal cortex BA9, Hippocampus, Hypothalamus, Brain nucleus accumbens basal ganglia, Brain putamen basal ganglia, Liver, Lung, Nerve tibial, Pituitary, Skin not Sun exposed suprapubic, Uterus
chr X: 15618061	*CLTRN*	34 / 46	Adipose subcutaneous, Adipose visceral omentum, Adrenal gland, Artery aorta, Brain amygdala, Brain anterior cingulate cortex BA24, Brain caudate basal ganglia, Brain cerebellar hemisphere, Brain cortex, Brain frontal cortex BA9, Hippocampus, Hypothalamus, Brain nucleus accumbens basal ganglia, Brain putamen basal ganglia, Mammary tissue, Transformed fibroblasts, Colon sigmoid, Colon transverse, Esophagus-gastroesophageal junction, Esophagus mucosa, Heart atrial appendage, Liver, Lung, Nerve tibial, Ovary, Pituitary, Prostate, Skin not Sun exposed suprapubic, Sun exposed lower leg, Small intestine terminal Ileum, Spleen, Thyroid, Uterus, Vagina

^#^represents the studied genetic variant in current study.

## Discussion

PE is a pregnancy-related syndrome linked to the death of more than 70,000 women and 500,000 fetuses worldwide each year (26, 27). Several studies have confirmed that PE is a complex disease caused by both environmental and genetic factors and carries a serious risk to the life and health of pregnant women and their fetuses (28, 29). Studies have shown that during normal pregnancy, ACE2 is secreted in abundance in the placenta and uterus, which represent important sources of the enzyme (30, 31). ACE2 exhibits high catalytic efficiency in the production of Ang-1–7, which is a vasodilator that inactivates the vasoconstrictor Ang II. This process contributes to systemic vasodilation and decreased blood pressure, as well as other physiological adaptations that occur during normal pregnancy. However, Ang-1–7 plasma levels are lower in pregnancies complicated by PE than in physiologically normal pregnancies (32). In addition, Song et al. (33) found a significant correlation between the *ACE2* rs879922 C>T variant and late-onset PE susceptibility in a Chinese population. These findings suggest that ACE2 plays an important regulatory role in the occurrence and development of PE.

In the current study, we explored the association between *ACE2* functional genetic variants (rs2106809 A>G, rs6632677 G>C, and rs2074192 C>T) and PE risk in a southern Chinese population. Demographic data showed that BMI, SBP, DBP, FPG, TG, TC, and HDL-C were much higher in PE cases than healthy controls, indicating that obesity, hypertension, hyperglycemia, and hyperlipidemia are involved in the pathogenesis of PE as risk factors. Analyses of associations between candidate SNPs and PE risk showed that the *ACE2* rs2106809 variant was significantly associated with the risk of PE, and this association was even more pronounced in subjects with higher pre-BMI (>23 kg/m^2^), TG (>2.84 mmol/L), and TC (>2.71 mmol/L) levels. Moreover, an interaction between rs2106809 and pre-BMI was also detected. This result was consistent with the results of comparisons of general data. It is likely that overweight or obesity before pregnancy or excessive weight gain during pregnancy can lead to abnormal metabolism, accompanied by fat accumulation in the body, thereby aggravating inflammation in placental blood vessels and trophoblasts resulting from the effects of genetic variants in initiating PE (34, 35). It can be seen from these data that rs2106809 might alter an individual’s genetic background or regulate the key clinical indicators of PE. Subgroup analyses often suffer from reduced statistical power or simply errors due to chance. Therefore, this finding needs to be confirmed in larger future studies.

Genetic variation often plays a minor role in PE, but the joint amplification effects of multiple variations can be captured. Recent studies have shown that the joint effects of multiple *ACE2* variants play a role in regulating blood pressure and dyslipidemia (9, 36), and the rs2074192 and rs2106809 haplotypes are associated with increased susceptibility to hypertension in women *via* a reduction in circulating Ang1–7 levels (37). Interestingly, although no significant association between rs6632677 G>C and rs2074192 C>T and PE risk was observed in the single-locus analysis, a complex gene–gene combination was detected in the MDR analysis. This result suggests that environmental factors, genetic factors, and combined environmental and genetic effects play a role in the pathogenesis of PE. These findings helped clarify the genetic component of *ACE2* in the risk of PE.

Studies have confirmed that genetic variants in the intron region of a gene can regulate gene expression and affect mRNA transcription levels (38, 39). It is speculated that the genetic variant rs2106809 A>G in the intron of *ACE2* affects the function of the ACE2 protein by modifying the transcription process of the gene, thereby ultimately affecting an individual’s susceptibility to PE. Another study showed that rs2106809 in *ACE2* may be a determinant of circulating Ang1–7 levels in female patients with essential hypertension (40). This could explain the mechanism by which rs2106809 alters susceptibility to PE by affecting the expression of *ACE2* and subsequent executive function of the ACE2 protein. Therefore, it is essential to explore the biological function of rs2106809 in future studies to verify its role in the pathogenesis of PE.

FPRP analysis is an effective method for determining the biological importance of associations (41). In this study, a relatively strict FPRP threshold of 0.2 was set. Among patients with pre-BMI >23 kg/m^2^, a significant correlation was observed between rs2106809 A>G and PE risk. This shows that these positive findings are possible, true, and reliable. In addition, the FPRP values obtained in some comparisons were much greater than 0.2, indicating that these important findings may have been observed by chance. Therefore, the conclusions drawn here are preliminary and need to be verified.

In view of the above findings, we explored the potential molecular mechanism of the significant association between rs2106809 A>G and PE risk and revealed the biological rationality of the observed association. As an intron variant, rs2106809 is thought to affect the post-transcriptional splicing of genes or regulate gene expression and affect mRNA transcription levels (38). The ASSP can be used to identify putative splice sites of genes and subsequently classify them as either constitutive or alternative isoform/cryptic splice sites using backpropagation networks (24). Subsequent analyses suggested some possible regulatory changes in putative splice sites due to the rs2106809 variant. Furthermore, eQTL analyses using the GTEx database, which incorporates 127 tissue/cell type-specific epigenome datasets, suggested that rs2106809 could regulate the expression of various functional genes (*PIR*, *ACE2*, and *CLTRN*) in certain tissues and cell lines. Studies of collectrin levels and PE onset showed that serum collectrin levels play a significant role in controlling blood pressure in pregnant women, with a significant inverse correlation between serum collectrin concentrations and blood pressure (42). The *PIR* gene is a transcriptional co-regulator of nuclear factor kappa-B (NF-κB), which is a tightly regulated molecule also related to inflammation and the control of innate and adaptive immune responses during onset of labor. Early activation of NF-κB may have an adverse effect by inducing premature termination of pregnancy, with bad outcomes for the mother and the fetus, including pregnancy loss (43). The above evidence reveals that as an eQTL, rs2106809 can affect physiologic processes such as immunity, glycolipid metabolism, and blood pressure by regulating the expression of functional genes and is therefore significantly associated with the pathogenesis of PE.

This study explored the relationship between functional variants of *ACE2* and the risk of PE, and etiological clues were provided from the perspective of genetics. An advantage of this study was its good design and application of multiple statistical analyses, which fully demonstrated the correlation between the studied *ACE2* gene variants and PE risk. However, this study still has some limitations. First, this was a hospital-based, case–control study and therefore might have selection bias. Second, although a relatively large sample was recruited for this study, the lower frequencies of some genotypes tested may still limit the efficiency of detecting significant associations in some subgroups. Third, this study did not experimentally explore the biological function of the significant association locus, and only functional prediction analysis was carried out using bioinformatics methods.

## Conclusion

The *ACE2* gene rs2106809 A>G variant is significantly associated with the risk of PE *via* individual locus effects and/or complex gene–gene and gene–environment interactions. Regulating the expression of functional genes such as *PIR*, *ACE2*, and *CLTRN* may be the molecular mechanism by which rs2106809 affects individual susceptibility to PE. A multi-center study with a larger sample size and functional experiments are needed to confirm these findings.

## Data availability statement

The original contributions presented in the study are publicly available. This data can be found here: https://doi.org/10.5061/dryad.crjdfn377.

## Ethics statement

This study was reviewed and approved by the Ethics Committee Review Board of Guilin Medical University and the Affiliated Hospital of Guilin Medical University. The patients/participants provided their written informed consent to participate in this study. Written informed consent was obtained from the individual(s) for the publication of any potentially identifiable images or data included in this article.

## Author contributions

XY: protocol/project development and manuscript editing. GH and YW: data collection and analysis, and manuscript writing. GH and LQ: data analysis and manuscript writing. BH: data collection and management. All authors contributed to the article and approved the submitted version.

## Funding

This study was supported by the Guangxi Young and middle-aged teachers’ basic ability improvement project (2020KY12028), Guangxi Natural Science Foundation of China (2020GXNSFAA238025), National Natural Science Foundation of China (81760614), the Key Research and Development Program (2018AB62004), and College Students’ Innovation Project (201810601031) of Guangxi, China.

## Conflict of interest

The authors declare that the research was conducted in the absence of any commercial or financial relationships that could be construed as a potential conflict of interest.

## Publisher’s note

All claims expressed in this article are solely those of the authors and do not necessarily represent those of their affiliated organizations, or those of the publisher, the editors and the reviewers. Any product that may be evaluated in this article, or claim that may be made by its manufacturer, is not guaranteed or endorsed by the publisher.
